# Ovarian Carcinoma With Isolated Spinal Cord Metastasis

**DOI:** 10.1177/2324709616657644

**Published:** 2016-07-08

**Authors:** Sarah Safadi, Patrick Rendon, Teresa Rutledge, Shadi Mayasy

**Affiliations:** 1 University of New Mexico Health Sciences Center, Albuquerque, NM, USA

**Keywords:** ovarian adenocarcinoma, metastasis to the spinal cord

## Abstract

Ovarian cancer metastasis to the spinal cord is quite rare, and few case reports have been published previously. Herein, we present a case of a patient who was treated for ovarian cancer and was thought to be disease free for 17 months, then presented with lower limb weakness. She was found to have a T11-T12 metastatic intramedullary spinal cord lesion. On pathology, the diagnosis of metastatic ovarian adenocarcinoma was made. This report highlights the importance of maintaining a low threshold for ovarian cancer metastases to the spinal cord when patients present with neurologic sequelae, even in the setting of normal laboratory values, as early detection can prevent permanent neurological consequences.

## Introduction

Ovarian cancer with metastasis is a well-known entity. Metastasis to the spinal cord, however, has rarely been documented with few reported cases in the literature. To our knowledge, only 6 previous case reports have identified ovarian cancer as the primary site of spinal metastasis.^1^

In this case report, we present the case of a patient who underwent treatment for ovarian cancer and was thought to be disease-free but presented with metastases to her spinal cord. For spinal cord metastasis, neurological status and the extent of damage prior to treatment is an important predictor of outcome, which depends on the extension of functional limitation, the tumor type, and the rapidity of onset of neurological deficits.^2^ This case highlights the importance of early detection of spinal metastases in ovarian cancer so that permanent neurological damage may be prevented for future patients.

## Case Presentation

A 78-year-old woman presented with a 2-week history of progressive lower limb weakness and bilateral numbness from her hips to her toes. Her primary care physician referred her for physical therapy, which did not improve her strength. Her symptoms continued to worsen until she became bed bound and presented to the hospital 2 months after the initial onset of symptoms. Her past medical history included stage IIIC ovarian adenocarcinoma 17 months prior to presentation. At that time, her Ca 125 level was 1612 units/mL. She received 6 cycles of neoadjuvant chemotherapy with carboplatin and Taxol, followed by exploratory laparotomy, bilateral salpingoopherectomy, splenectomy (due to concerning lesions in the spleen), and 3 cycles of chemotherapy. Two subsequent abdominal computed tomography (CT) scans showed no evidence of recurrence and Ca 125 levels had decreased to normal.

**Figure 1.**
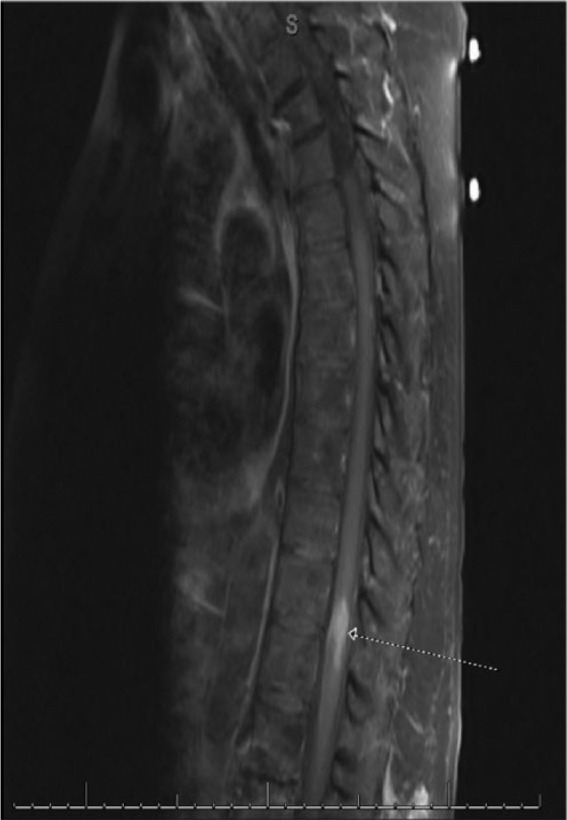
Sagittal spinal MRI shows an enhancing intramedullary lesion of the thoracic spinal cord at T11-T12.

**Figure 2.**
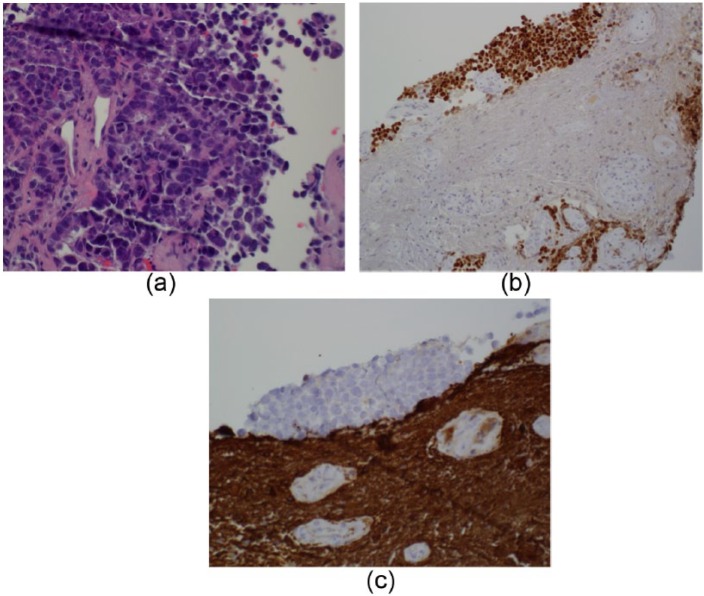
(a) Biopsy results showing tumor cells (H&C; 40×). (b) Biopsy slide (20×) done using Pax 8 stain, which supports the diagnosis of ovarian adenocarcinoma. Tumor cell nuclei are positive. (c) Biopsy slide (40×) done with GFAP stain, which is positive for glial cells. Tumor cells are negative.

On exam she had left lower extremity weakness; 2/5 in both hip and knee flexion and extension, 4/5 in dorsiflexion and plantar flexion. Right lower extremity strength was 0/5 in both hip and knee flexion and extension, and 3/5 in dorsiflexion and plantar flexion. Ca 125 levels were normal. Cerebrospinal fluid analysis indicated nonspecific inflammatory changes and no sign of infection. Spinal magnetic resonance imaging (MRI) showed an intramedullary lesion at T11-T12 (Figure 1). Positron emission tomography CT revealed focally increased FDG avidity at T11-T12. A laminectomy and biopsy of the lesion were performed; pathology indicated metastatic ovarian cancer (Figure 2a-c).

The patient received high-dose corticosteroid therapy for 1 week after her presentation. She was discharged to a rehabilitation facility and underwent outpatient radiation therapy with 30 Gy in 10 fractions of 300 cGy each, from T7 to L1. She tolerated the treatment well and gained slight improvement in sensation and weakness, but was unable to ambulate. She received a subsequent 6 cycles of chemotherapy with Taxol and carboplatin. On completion of this chemotherapy, her weakness improved with motor strength in the left lower extremity 4/5, and in the right lower extremity 3/5 in all muscle groups. MRI of the thoracic spine 4 weeks after the last cycle showed widespread bony metastases but no spinal cord compression. An MRI of the cervical spine showed no enhancing lesions. A CT of the abdomen and pelvis was without masses in the pelvis. Ultimately, patient gained improvement in her lower extremity strength, but she did not regain full function.

## Discussion

Intramedullary spinal cord metastasis is quite rare, with a reported prevalence of up to 2.1% in an autopsy series of patients with cancer.^3^ Patients most commonly present with sensory deficits (40% to 90%) and pain (83% to 95%), with weakness (60% to 85%) and autonomic dysfunction (40% to 57%) being less common.^2^

The mechanism of metastatic spread for ovarian cancer may explain this phenomenon. Ovarian cancer does not exhibit the classic hematogenous spread found in most other cancers. Studies suggest that serous ovarian carcinomas grow very efficiently within the peritoneal cavity, but rarely metastasize outside of the area. When metastasis does occur, the hypothesized process states that as tumor cells detach singularly or in clusters from the primary tumor, they then travel by peritoneal fluid into the peritoneum and omentum.^4^

Distant metastases have been reported to the liver, brain, and other sites. Clinical predictors for distant metastasis remain unclear. Possible correlations are P53 mutations, genomic instability, and vascularization of the tumor. One of the few studies regarding distant metastasis in ovarian cancer studied 130 patients with ovarian cancer. Twenty-two percent were found to have distant metastasis either on initial presentation or during the course of the treatment. Multiple variables were considered as possible predictors of distant metastasis, including p53 null mutation; high-stage, high-grade, nodal metastasis; and presence of ascites. P53 null mutation was the most significant of these, followed by stage.^5^

As reported prior, spinal cord metastasis from ovarian cancer portends a poor prognosis with a survival of 10 months to 3 years.^1^ There are several factors that may play a role in the varied prognoses of such patients. This includes time of symptom onset, diagnosis delays, and treatment course. Thus, a better understanding of this disease pattern and early recognition of metastasis may prevent delays in diagnosis and permanent neurological damage. Prior case reports have not reported if there was a delay in diagnosis, and whether that lead to further permanent neurological damage. For our patient, the diagnosis was delayed for 3 months, and the treatment she received did ultimately improve lower extremity strength, but she did not regain full function. It is likely because the rarity of this condition may have contributed to the initial referral for physical therapy rather than establishing workup and proceeding with imaging studies. On her current presentation, the normal CA 125 level was misleading as well, and likely lead to increased time spent on exclusion of other diagnoses and performance of unnecessary tests such as lumbar puncture. Other published cases also reported normal Ca 125 levels, and no diagnostic value of lumbar puncture.^1^

Specialized attention to patients with history of ovarian cancer and neurologic sequelae, including a detailed neurological exam or spinal MRI, may be employed in the future as screening measures for the benefit of patients with this uncommon condition.
